# Target Identification of the Marine Natural Products Dictyoceratin-A and -C as Selective Growth Inhibitors in Cancer Cells Adapted to Hypoxic Environments

**DOI:** 10.3390/md17030163

**Published:** 2019-03-08

**Authors:** Takashi Kawachi, Shun Tanaka, Akinori Fukuda, Yuji Sumii, Andi Setiawan, Naoyuki Kotoku, Motomasa Kobayashi, Masayoshi Arai

**Affiliations:** 1 Graduate School of Pharmaceutical Sciences, Osaka University, 1-6 Yamadaoka, Suita, Osaka 565-0871, Japan; pabro-ai@hotmail.co.jp (T.K.); tanaka.eclipse@gmail.com (S.T.); sukoshi1293fushigi@yahoo.co.jp (A.F.); sumii.yuji@nitech.ac.jp (Y.S.); kotoku@fc.ritsumei.ac.jp (N.K.); kobayasi@phs.osaka-u.ac.jp (M.K.); 2 Department of Chemistry, Faculty of Science, Lampung University, J1. Prof. Dr. Sumantri Brodjonegoro No. 1, Bandar Lampung 35145, Indonesia; asetiawan0922@gmail.com

**Keywords:** dictyoceratin-A, hypoxia, cancer, RNA polymerase II-associated protein 3, probe molecule, target molecule

## Abstract

Hypoxia-adapted cancer cells in tumors contribute to the pathological progression of cancer. The marine spongean sesquiterpene phenols dictyoceratin-A (**1**) and -C (**2**) have been shown to induce hypoxia-selective growth inhibition in cultured cancer cells and exhibit in vivo antitumor effects. These compounds inhibit the accumulation of hypoxia-inducible factor-1α (HIF-1α), which is a drug target in hypoxia-adapted cancer cells, under hypoxic conditions. However, the target molecules of compounds **1** and **2**, which are responsible for decreasing HIF-1α expression under hypoxic conditions, remain unclear. In this study, we synthesized probe molecules for compounds **1** and **2** to identify their target molecules and found that both compounds bind to RNA polymerase II-associated protein 3 (RPAP3), which is a component of the R2TP/Prefoldin-like (PEDL) complex. In addition, RPAP3-knockdown cells showed a phenotype similar to that of compound-treated cells.

## 1. Introduction

In tumors, the hypoxic environment is an important factor for tumor growth, angiogenesis, metastasis, and response to chemotherapy and irradiation. Tumor cells in the hypoxic environment show resistance to chemotherapy and irradiation and enhancement of angiogenesis [1]. In addition, the hypoxic environment in a tumor is unlike that in normal tissues. Therefore, compounds that selectively inhibit the growth of tumor cells under hypoxic conditions are expected to have potential applications as anticancer drugs. The heterodimeric transcription factor hypoxia-inducible factor (HIF)-1, which comprises an oxygen-regulated α-subunit and a constitutively expressed β-subunit, is a well-studied regulator of cellular response to hypoxia. Decreased HIF-1 activity is usually associated with a slower growing and less angiogenic tumor phenotype [2]. Therefore, HIF-1, particularly HIF-1α, has been extensively studied as a potential drug target for cancer chemotherapy.

The marine environment is a rich source of drug leads because of its chemical and biological diversity. Indeed, some compounds, including ampelopsin [3], salternamide A [4], eudistidine A [5], discorhabdins [6], laurenditerpenol [7], and strongylophorines [8], have been found to inhibit HIF-1α or its signaling pathway. To date, we have also screened the extracts of marine organisms to discover bioactive natural products showing selective growth inhibition in hypoxia-adapted DU145 human prostate cancer cells. We rediscovered furospinosulin-1, a furanosesterterpene from the Indonesian marine sponge *Dactylospongia elegans*, by bioassay-guided separation [9]. Furospinosulin-1 has been shown to cause hypoxia-selective growth inhibition in cultured cancer cells and exhibits in vivo antitumor effects. A target identification study revealed that furospinosulin-1 directly binds to the transcriptional regulators p54^nrb^ and lens epithelium-derived growth factor/p75 [10]. In addition, further investigations of the constituents of *D. elegans* extracts inducing hypoxia-selective growth inhibition have led to the isolation of sesquiterpene phenol dictyoceratin-C (**2**) as an active substance and have demonstrated that dictyoceratin-A (**1**) shows similar biological activity [11]. We then achieved the total synthesis of compounds **1** and **2** and clarified that these compounds show potent antitumor activity in mice inoculated with mouse sarcoma S180 cells by oral administration [12,13]. Analysis of the mode of action revealed that compounds **1** and **2** inhibit the accumulation of HIF-1α in hypoxia-adapted DU145 cells [11]. Therefore, hypoxia-selective growth inhibition of cancer cells by treatment with compounds **1** and **2** may result from decreased HIF-1α accumulation under hypoxic conditions. However, the detailed mechanisms of action and target molecules of compounds **1** and **2**, which regulate HIF-1α expression, have not been identified.

Accordingly, in this study, we synthesized probe molecules to analyze the binding proteins of compounds **1** and **2** based on structure-activity relationships using synthetic analogs of the compounds [13]. We then characterized the mechanisms through which the compounds modulate cancer cells. Our findings provide important insights into the applications of dictyoceratin-A (**1**) and -C (**2**) as candidate drugs in the treatment of cancer.

## 2. Results and Discussion

### 2.1. Effects of Probe Molecules on the Growth of DU145 Cells under Normoxic and Hypoxic Conditions

**Figure 1:**
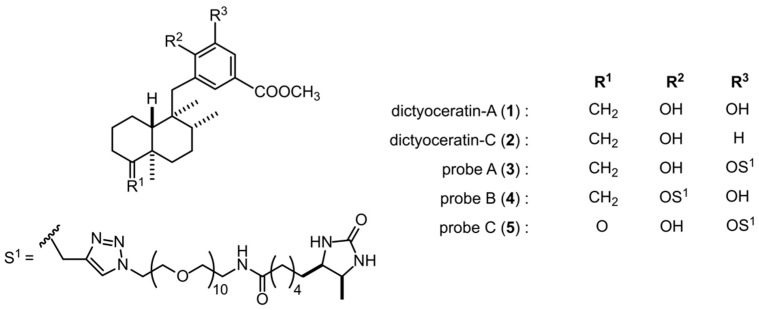
Chemical structures of dictyoceratin-A (**1**) and -C (**2**) and their probes (**3**–**5**).

**Figure 2:**
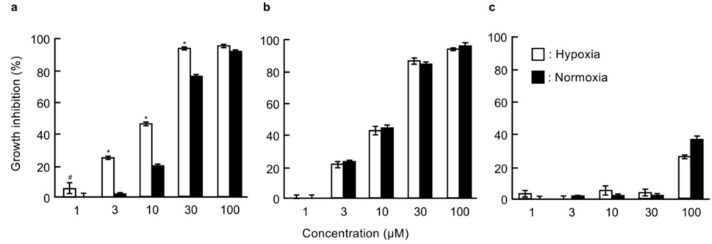
Growth inhibitory activities of probes **3**–**5** in DU145 cells under normoxic and hypoxic conditions. DU145 cells (1 × 10^4^ cells/well/200 µL) in 96-well plates were pre-incubated for 12 h under normoxic or hypoxic conditions. The cells were then treated with the indicated concentrations of probe A (**3**, **a**), probe B (**4**, **b**), or probe C (**5**, **c**) for 24 h under normoxic or hypoxic conditions. The growth inhibition rate was calculated as the percentage of parallel negative controls. Differences were considered significant at * *p* < 0.01 and ^#^ *p* < 0.05.

In order to identify the target molecules of dictyoceratin-A (**1**) and -C (**2**) as selective growth inhibitors of cancer cells adapted to hypoxic environments, we synthesized three types of probe molecules (**3**–**5**) based on an analysis of structure-activity relationships using synthetic analogs of **1** and **2** (Figure 1 and Scheme S1) [13]. As shown in Figure 2a, probe A (**3**) induced selective growth inhibition in DU145 cells cultured under hypoxic conditions. In contrast, probe B (**4**) induced growth inhibition in DU145 cells, but showed no selectivity between normoxic and hypoxic conditions (Figure 2b). In addition, probe C (**5**) did not exhibit growth inhibitory activity in DU145 cells (Figure 2c). We then performed target identification for dictyoceratin-A (**1**) and -C (**2**) using probes showing different biological activities in DU145 cells.

### 2.2. Analysis of Target Molecules Using Probe A (**3**) from a Peptide-Displayed Phage Library

We constructed a peptide-displayed phage library from mRNA extracted from DU145 cells cultured under hypoxic conditions. The binding protein for **1** and **2** was then investigated by phage display using probe A (**3**) [14]. After seven rounds of biopanning, 30 clones of phages that bound to probe A (**3**) by interacting with the displayed peptide were randomly selected, and we then analyzed the DNA sequences in each phage to clarify the displayed peptide. The obtained partial peptides of proteins were then displayed on the phages that bound to probe A (**3**), as follows: RNA-binding protein 28 (RBM28, UniProt ID: Q9NW13) from five phages, RNA polymerase II-associated protein 3 (RPAP3, UniProt ID: Q9H6T3) from three phages, melanoma inhibitory activity protein 3 (MIA3, UniProt ID: Q5JRA6) from two phages, eukaryotic translation initiation factor 5A-1-like (EIF5AL1, UniProt ID: Q6IS14) from two phages, tRNA (adenine(58)-*N*(1))-methyltransferase noncatalytic subunit TRM6 (TRMT6, UniProt ID: Q9UJA5) from two phages, NADH-ubiquinone oxidoreductase chain 4 (UniProt ID: P03905) from one phage, quinone oxidoreductase (UniProt ID: Q08257) from one phage, l-lactate dehydrogenase A chain (UniProt ID: P00338) from one phage, and six types of ribosomal proteins, including 40S ribosomal protein S3 (RPS3, UniProt ID: P23396) and 60S ribosomal protein L28 (RPL28, UniProt ID: P46779), from 13 phages. Among these targets, we speculated that the proteins other than the six ribosomal proteins were candidate binding proteins for dictyoceratin-A (**1**) and -C (**2**) because ribosomal proteins are often selected as nonspecific binding proteins in studies of target identification.

### 2.3. Binding Selectivity of the Selected Peptide-Displayed Phages against Probes A–C (**3–5**)

**Figure 3:**
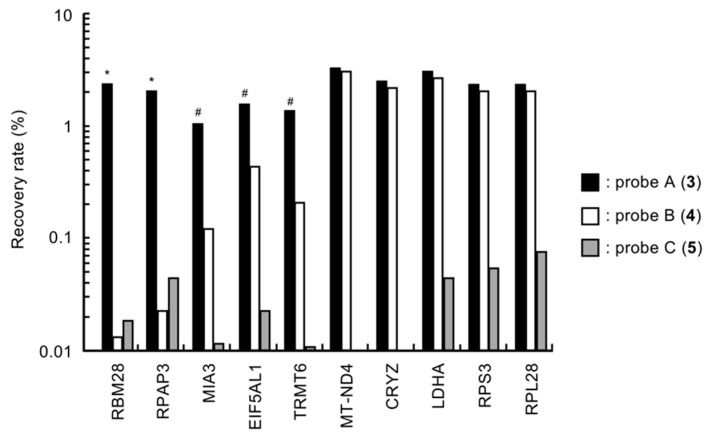
Binding selectivity of the candidate phages for probes **3**–**5**. The binding abilities of probes **3**–**5** to the phages were evaluated according to the recovery rates of the phages. Each phage solution (1 × 10^9^ pfu/mL) was mixed with 200 pmol probe, and a single cycle of biopanning was performed. Then, the plaque-forming units (titers) of the eluate from Dynabeads were measured, and the recovery rate was calculated. Differences were considered significant at * *p* < 0.01 and ^#^ *p* < 0.05.

To confirm candidate binding proteins of compounds **1** and **2**, the binding selectivity of each phage against probes A–C (**3**–**5**) was investigated. In this experiment, phages displaying partial peptides of RPS3 and RPL28 were used as representative examples of ribosomal proteins. The recovery rate of each phage was used as an index of binding ability of the probes to each phage. As shown in Figure 3, probe C (**5**), which did not exhibit growth inhibitory activity against DU145 cells, showed lower binding abilities to all phages. Indeed, the recovery rates of phages were less than 0.06%. Phages displaying the partial peptides of RBM28 and RPAP3 bound selectively to probe A (**3**) that showed selective growth inhibition in hypoxia-adapted DU145 cells. In contrast, the recovery rates of phages displaying partial peptides of MIA3, EIF5AL1, and TRMT6 against probe A (**3**) were all more than 1%, and those of these phages against probe B (**4**) ranged from 0.1% to 0.4%. This result indicated that these phages bound to both probes with moderate selectivity for probe A (**3**). In addition, another five phages bound to both probe A (**3**) and probe B (**4**), with similar binding abilities. Therefore, we speculated that five proteins, i.e., RBM28, RPAP3, MIA3, EIF5AL1, and TRMT6, were candidate binding proteins of **1** and **2**. 

### 2.4. Binding Abilities of Probe A (3) with RBM28, RPAP3, MIA3, EIF5AL1, and TRMT6 in CELL lysates

**Figure 4:**
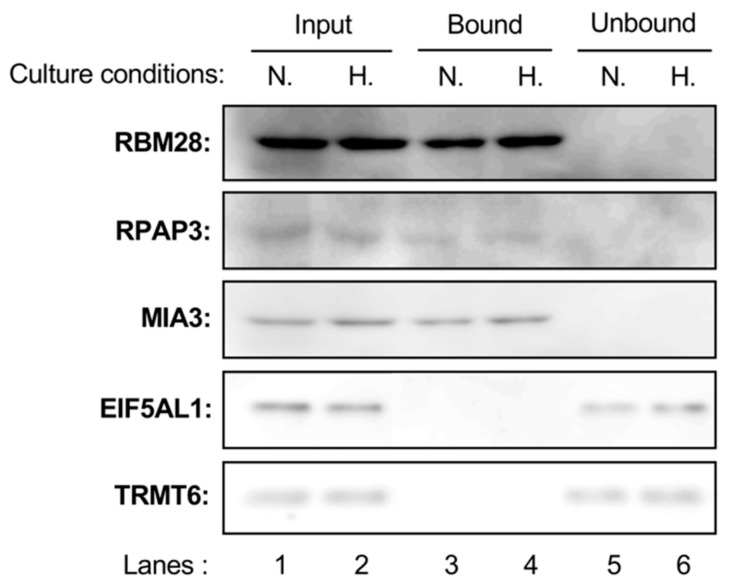
Binding of probe A (**3**) to the candidate proteins in cell lysates. Cell lysates (100 μg protein each), prepared from DU145 cells cultured under hypoxic conditions (H, lanes 2, 4, and 6) or normoxic conditions (N, lanes 1, 3, and 5) were incubated with probe A (**3**). The probe with bound protein was then separated from the supernatant by the addition of streptavidin-conjugated Dynabeads. The resulting supernatants and Dynabeads were used for western blotting analysis to detect RBM28, RPAP3, MIA3, EIF5AL1, and TRMT6.

Next, we investigated whether probe A (**3**) bound to RBM28, RPAP3, MIA3, EIF5AL1, and TRMT6 in cell lysates (Figure 4). As shown in lanes 1 and 2, the expression levels of each protein in DU145 cells were not different between hypoxic and normoxic conditions. Probe A (**3**) was found to bind to RBM28, RPAP3, and MIA3 in cell lysates prepared from DU145 cells cultured under both hypoxic and normoxic conditions (lanes 3 and 4), whereas EIF5AL1 and TRMT6 in cell lysates did not bind with probe A (**3**) (lanes 5 and 6). This result suggests that RBM28, RPAP3, and MIA3 may be binding proteins of **1** and **2**.

### 2.5. Growth of RBM28-, RPAP3-, and MIA3-Knockdown DU145 Cells under Normoxic and Hypoxic Conditions

**Figure 5:**
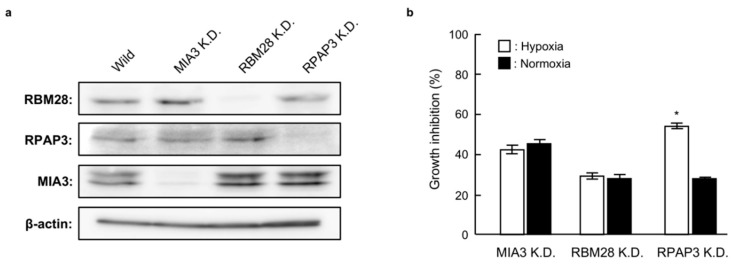
Growth of RBM28-, RPAP3-, and MIA30-knockdown DU145 cells under normoxic and hypoxic conditions. (**a**) The expression levels of the indicated proteins in each knockdown cell line were analyzed by western blotting. The expression level of each protein was calculated from its relative density, which was normalized to that of β-actin. (**b**) The proliferation rates of MIA3-, RBM28-, and RPAP3-knockdown DU145 cells were investigated under normoxic and hypoxic conditions. The growth inhibition rate was calculated as a percentage relative to the negative control cells. Differences were considered significant at * *p* < 0.01.

In order to identify the binding proteins of **1** and **2**, we investigated the effects of RBM28, RPAP3, and MIA3 knockdown on the growth of DU145 cells. As shown in Figure 5a, small interfering RNA (siRNA) targeting RBM28, RPAP3, and MIA3 reduced the levels of the corresponding proteins compared with that in wild-type DU145 cells. Among these knockdown cells, RPAP3-knockdown cells showed decreased growth under hypoxic conditions compared with normoxic conditions; no selectivity was observed for MIA3- or RBM28-knockdown cells. The phenotype of RPAP3-knockdown cells is the same as compound-treated cells [11]. Therefore, this result strongly suggests that RPAP3 was a binding protein for **1** and **2**.

### 2.6. Characterization of RPAP3-Knockdown DU145 Cells

**Figure 6:**
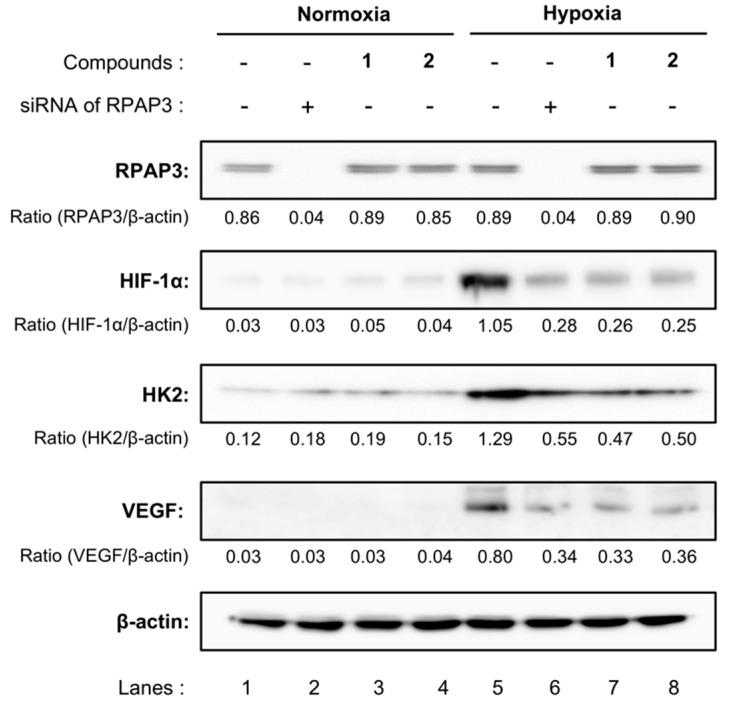
Expression of hypoxia-related proteins in RPAP3-knockdown DU145 cells. The expression levels of the indicated hypoxia-related proteins in RPAP3-knockdown DU145 cells were compared with those of wild-type DU145 cells treated with/without dictyoceratin-A (**1**) or -C (**2**) (10 µM each) using western blotting analysis. The expression level of each protein was calculated from its relative density, which was normalized to that of β-actin.

We further compared the expression of HIF-1α and related proteins in RPAP3-knockdown DU145 cells with those of DU145 cells treated with compound **1** or **2** (Figure 6). The expression levels of HIF-1α in DU145 cells were elevated under hypoxic conditions. Consistent with these findings, the expression levels of hexokinase 2 (HK2) and vascular endothelial growth factor (VEGF), which are regulated by HIF-1α, also increased by cultivating DU145 cells under hypoxic conditions (lanes 1 and 5). Treatment with dictyoceratin-A (**1**) or -C (**2**) reduced the expression levels of HIF-1α, HK2, and VEGF (lanes 3, 4, 7, and 8), as described previously [11]. Moreover, protein expression levels of RPAP3-knockdown cells were similar to those in **1**- or **2**-treated DU145 cells. Indeed, RPAP3-knockdown cells showed reduced HIF-1α, HK2, and VEGF expression (lanes 2 and 6).

### 2.7. Effects of RPAP3 Overexpression on the Growth Inhibitory Activity of Dictyoceratin-A (**1**)

**Figure 7:**
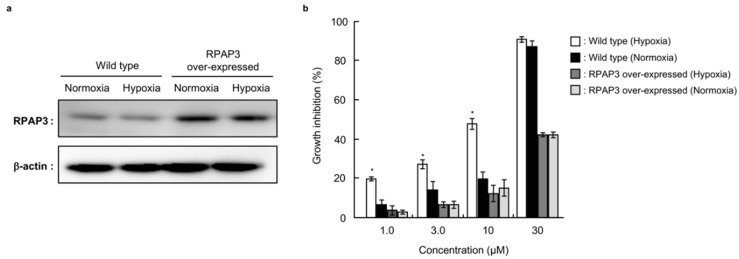
Effect of dictyoceratin-A (**1**) on RPAP3-overexpressing DU145 cells. (**a**) The expression levels of RPAP3 protein in stable RPAP3-overexpressing cells were compared with those in wild-type DU145 cells using western blotting. The expression levels of RPAP3 were calculated based on the relative density of bands, normalized to that of β-actin. (**b**) Antiproliferative activity of dictyoceratin-A (**1**) in wild-type and RPAP3-overexpressing DU145 cells. The growth inhibition rate was calculated as a percentage relative to that in control cells. Differences were considered significant at * *p* < 0.01.

In order to investigate whether dictyoceratin-A (**1**) and -C (**2**) interacted with RPAP3 in DU145 cells, we investigated the effects of dictyoceratin-A (**1**) on the growth of RPAP3-overexpressing DU145 cells (Figure 7). As shown in Figure 7a, the expression levels of RPAP3 protein in the generated RPAP3-overexpressing DU145 cells were increased 3.4-fold compared with that in wild-type DU145 cells. While dictyoceratin-A (**1**) induced hypoxia-selective growth inhibition in wild-type of DU145 cells, RPAP3-overexpressing DU145 cells showed resistance to compound **1** to the levels of normoxic conditions (Figure 7b). This result indicates that dictyoceratin-A (**1**) interacted with RPAP3 in DU145 cells.

**Figure 8:**
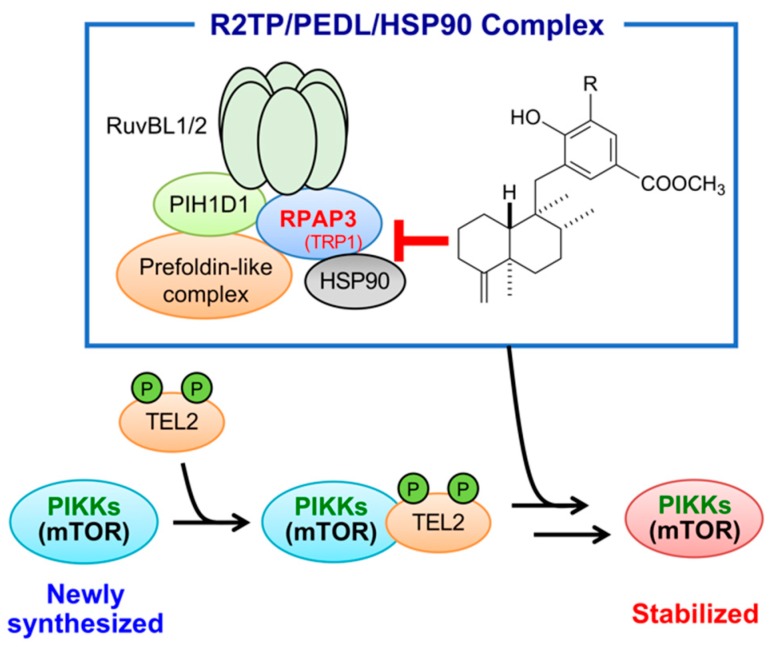
Structure and function of R2TP/PEDL/HSP90 complex, and target of dictyoceratin-A (**1**) and -C (**2**) as hypoxia-selective growth inhibitors in cancer cells.

RPAP3 is a component of the R2TP/Prefoldin-like (PEDL) complex, which facilitates the assembly, activation, and cellular stability of RNA polymerase II, phosphatidylinositol 3-kinase-related kinases (PIKKs), and small nuclear ribonuclear proteins [15,16,17]. The R2TP/PEDL complex collaborates with heat-shock protein 90 (HSP90) to facilitate its function; RPAP3 is known to interact with HSP90 at the tetratricopeptide repeat 1 (amino acids 133–234) and 2 (amino acids 282–383) domains of RPAP3 [18,19]. Gene sequencing in probe A (**3**)-binding phages indicated that the phages displayed the region of RPAP3 protein containing amino acids 109–231, which is close to the TRP1 domain. Therefore, dictyoceratin-A (**1**) and -C (**2**) might inhibit the interaction between RPAP3 and HSP90. PIKKs include various proteins related to DNA damage signaling (e.g., ATM and ATR), mRNA surveillance (e.g., SMG1), transcriptional regulation (e.g., transport protein particle), and cell metabolism signaling (e.g., mammalian target of rapamycin [mTOR]) [20]. Among these proteins, mTOR is known to be involved in the regulation of HIF-1α expression [21]. Moreover, newly synthesized mTOR is associated with phosphorylated Tel2 protein, and the mTOR/Tel2 complex interacts with the R2TP/PEDL/HSP90 complex to stabilize mTOR [22,23] (Figure 8).

Further studies are needed to assess the affinity of compounds with RPAP3, the structure of the R2TP/PEDL/HSP90 complex and signaling of mTOR after treatment with compounds. However, our current findings suggested that dictyoceratin-A (**1**) and -C (**2**) bound to RPAP3 in the vicinity of the TRP1 domain and disrupted the R2TP/PEDL/HSP90 complex, leading to dysfunction of mTOR and reduced accumulation of HIF-1α. These factors may have resulted in growth inhibition in DU145 cells under hypoxic conditions (Figure 8).

## 3. Materials and Methods 

### 3.1. General Reagents and Materials

RPMI 1640 medium was purchased from Nacalai Tesque, Inc. (Kyoto, Japan). Fetal bovine serum (FBS; lot 30-2215) was purchased from Equitech-Bio Inc. (Kerrville, TX, USA). Monoclonal mouse anti-HIF-1α antibodies were obtained from R&D Systems, Inc. (Minneapolis, MN, USA). Monoclonal mouse anti-VEGF antibodies were purchased from Abnova (Taipei, Taiwan). Polyclonal rabbit anti-MIA3 and anti-TRMT6 antibodies were obtained from Abcam (Cambridge, UK). Polyclonal rabbit anti-EIF5AL1 and anti-RBM28 antibodies were obtained from Abgent Inc. (San Diego, CA, USA). Polyclonal rabbit anti-β-actin and monoclonal rabbit anti-HK2 antibodies were purchased from Cell Signaling Technology, Inc. (Danvers, MA, USA). Polyclonal rabbit anti-RPAP3 antibodies were obtained from Bethyl Laboratories, Inc. (Montgomery, TX, USA). Horseradish peroxidase (HRP)-linked anti-mouse IgG and anti-rabbit IgG antibodies (GE Healthcare Life Sciences, Buckinghamshire, UK) were used as secondary antibodies. Streptavidin-conjugated Dynabeads (M-280) were purchased from Invitrogen (Carlsbad, CA, USA). G418 was obtained from Wako Pure Chemical Industries, Ltd. (Osaka, Japan). DNA restriction enzymes and T4 DNA ligase were obtained from New England BioLabs Inc. (Ipswich, MA, USA). The Expand High Fidelity PCR System (Roche Applied Science, Mannheim, Germany) was used for polymerase chain reaction. Kanamycin, 3-(4,5-dimethylthiazol-2-yl)-2,5-diphenyltetrazolium bromide (MTT), and other chemicals were purchased from Sigma-Aldrich (St. Louis, MO, USA) or Kishida Chemical Co., Ltd. (Osaka, Japan).

### 3.2. Cell Culture and Antiproliferative Activity

Human DU145 prostate cancer cells and their transformants were maintained in RPMI 1640 supplemented with heat-inactivated 10% FBS and kanamycin (50 μg/mL) in a humidified atmosphere containing 5% CO_2_ at 37 °C. For analysis of antiproliferative activity, cells in culture medium were plated in 96-well plates (1 × 10^4^ cells/200 μL/well) for 4 h in a humidified atmosphere containing 5% CO_2_ at 37 °C (normoxic conditions). Next, the plates were incubated for 12 h in 94% N_2_, 5% CO_2_, and 1% O_2_ to induce hypoxia-related genes, such as HIF-1α (hypoxic conditions) or in a humidified atmosphere of 5% CO_2_ at 37 °C (normoxic conditions). After incubation, test compounds were added, and the plates were incubated for an additional 24 h under the same conditions. Cell proliferation was detected according to an established MTT method as previously described [24]. The growth inhibition rate was calculated as a percentage relative to negative controls.

### 3.3. Synthesis of Probe Molecules 

#### 3.3.1. General Instruments

The following instruments were used to obtain physical data: a JEOL ECA-500 (^1^H-NMR: 500 MHz, ^13^C-NMR: 125 MHz) spectrometer (JEOL Ltd., Tokyo, Japan) for ^1^H and ^13^C NMR data using tetramethylsilane or residual solvent peak as internal standards; a JASCO FT/IR-5300 infrared spectrometer (JASCO Corp., Tokyo, Japan) for IR spectra; a Waters Q-Tof Ultima API mass spectrometer (Waters Corp., Milford, MA, USA) for ESI-TOF MS. Silica gel (40–100 μm, Kanto Chemical Co. Inc., Tokyo, Japan ) and pre-coated thin layer chromatography (TLC) plates (Merck, 60F_254_, Darmstadt, Germany) were used for column chromatography and TLC. Spots on TLC plates were detected by spraying acidic p-anisaldehyde solution (p-anisaldehyde: 25 mL, c-H_2_SO_4_: 25 mL, AcOH: 5 mL, EtOH: 425 mL) or phosphomolybdic acid solution (phosphomolybdic acid: 25 g, EtOH: 500 mL) with subsequent heating. Unless otherwise noted, all reactions were performed under N_2_ atmosphere. Compounds **7**, **8**, and **9** were synthesized according to our previous report [13].

#### 3.3.2. *N*-(32-Azido-3,6,9,12,15,18,21,24,27,30-decaoxadotriacontyl)-6-((4*R*,5*S*)-5-methyl-2-oxoimidazolidin-4-yl)hexanamide (**6**)

To a solution of desthiobiotin (68.7 mg, 0.321 mmol), which has similar properties as biotin [25], and *O*-(2-aminoethyl)-*O*’-(2-azidoethyl)nonaethylene glycol (161 mg, 0.301 mmol) in DMF (3.0 mL) was added EDCI·HCl (67.3 mg, 0.353 mmol) and HOBt (47.7 mg, 0.353 mmol), and the whole mixture was stirred for 20 h at rt. Concentration in vacuo gave a crude product, which was purified by SiO_2_ column chromatography (CHCl_3_/MeOH/H_2_O = 30:3:1, lower phase) to give **6** (88.4 mg, 40%) as colorless amorphous solid.

^1^H-NMR (500 MHz, CDCl_3_) δ: 6.55 (1H, t, *J* = 4.9 Hz), 5.52 (1H, s), 4.80 (1H, s), 3.83-3.79 (1H, m), 3.70–3.59 (39H, m), 3.55 (2H, t, *J* = 4.9 Hz), 3.42 (2H, q, *J* = 5.2 Hz), 3.38 (2H, t, *J* = 5.2 Hz), 2.17 (2H, t, *J* = 7.2 Hz), 1.67–1.62 (2H, m), 1.50–1.23 (6H, m), 1.10 (3H, d, *J* = 6.3 Hz). ^13^C-NMR (125 MHz, CDCl_3_) δ: 173.0, 163.5, 70.63, 70.60, 70.57, 70.49 (12C), 70.42, 70.37, 70.1, 70.0, 69.9, 56.0, 51.3, 50.6, 39.1, 35.9, 29.4, 28.6, 25.9, 25.1, 15.7. IR (KBr): 3249, 2867, 2104, 1105 cm^−1^. MS (ESI-TOF) *m*/*z*: 745 [M + Na]^+^. HRMS (ESI-TOF) *m*/*z*: 745.4323 calcd for C_32_H_62_N_6_O_12_Na; Found: 745.4355.

#### 3.3.3. Methyl 4-Hydroxy-3-((1-(39-((4*R*,5*S*)-5-methyl-2-oxoimidazolidin-4-yl)-34-oxo-3,6,9,12,15,18,21,24,27,30-decaoxa-33-azanonatriacontyl)-1*H*-1,2,3-triazol-4-yl)methoxy)-5-(((1*S*,2*R*,4a*R*,8a*R*)-1,2,4a-trimethyl-5-methylenedecahydronaphthalen-1-yl)methyl)benzoate (**3**, probe A)

To a solution of **7** (2.5 mg, 6.09 μmol) and **6** (6.6 mg, 9.74 μmol) in DMF (0.2 mL) was added CuI (0.3 mg, 1.83 μmol) and DIPEA (1.6 μL, 9.13 μmol), and the whole mixture was stirred for 16 h at rt. Concentration in vacuo gave a crude product, which was purified by SiO_2_ column chromatography (CHCl_3_/MeOH/H_2_O = 15:3:1, lower phase) to give **3** (6.9 mg, quantitative yield) as colorless amorphous solid.

^1^H-NMR (500 MHz, CDCl_3_) δ: 7.94 (1H, s), 7.54 (1H, d, *J* = 1.7 Hz), 7.47 (1H, d, *J* = 1.7 Hz), 6.80 (1H, s), 6.33 (1H, br s), 5.25 (2H, s), 4.71 (1H, br s), 4.58 (2H, t, *J* = 4.9 Hz), 4.40 (1H, s), 4.36 (1H, s), 4.33 (1H, br s), 3.89-3.88 (2H, m), 3.87 (3H, s), 3.83 (1H, t, *J* = 6.9 Hz), 3.71–3.60 (34H, m), 3.55 (2H, t, *J* = 4.9 Hz), 3.44 (2H, dd, *J* = 10.0, 5.4 Hz), 2.70 (1H, d, *J* = 13.7 Hz), 2.63 (1H, d, *J* = 13.7 Hz), 2.33 (1H, td, *J* = 13.7, 4.8 Hz), 2.18 (2H, t, *J* = 7.4 Hz), 2.09 (2H, br s), 1.91 (1H, d, *J* = 12.6 Hz), 1.65 (2H, t, J = 7.4 Hz), 1.59–1.17 (16H, m), 1.12 (3H, d, *J* = 6.9 Hz), 1.05 (3H, s), 1.01 (3H, d, *J* = 6.3 Hz), 0.94 (1H, dd, *J* = 12.0, 1.7 Hz), 0.86 (3H, s). MS (ESI-TOF) *m*/*z*: 1155 [M + Na]^+^. HRMS (ESI-TOF) *m*/*z*: 1155.6781 calcd for C_58_H_96_N_6_O_16_Na; Found: 1155.6814.

#### 3.3.4. Methyl 3-hydroxy-4-((1-(39-((4*R*,5*S*)-5-methyl-2-oxoimidazolidin-4-yl)-34-oxo-3,6,9,12,15,18,21,24,27,30-decaoxa-33-azanonatriacontyl)-1*H*-1,2,3-triazol-4-yl)methoxy)-5-(((1*S*,2*R*,4a*R*,8a*R*)-1,2,4a-trimethyl-5-methylenedecahydronaphthalen-1-yl)methyl)benzoate (**4**, probe B)

To a solution of **8** (3.6 mg, 8.77 μmol) and **6** (9.5 mg, 13.2 μmol) in DMF (0.2 mL) was added CuI (0.5 mg, 2.63 μmol) and DIPEA (2.3 μL, 13.2 μmol), and the whole mixture was stirred for 16 h at rt. Concentration in vacuo gave a crude product, which was purified by SiO_2_ column chromatography (CHCl_3_/MeOH/H_2_O = 15:3:1, lower phase) to give **4** (9.8 mg, 98%) as colorless amorphous solid.

^1^H-NMR (500 MHz, CDCl_3_) δ: 7.83 (1H, br s), 7.48 (1H, d, *J* = 2.3 Hz), 7.32 (1H, d, *J* = 2.3 Hz), 6.47 (1H, br s), 5.14 (1H, d, *J* = 11.8 Hz), 5.01 (1H, d, *J* = 11.8 Hz), 4.56 (3H, br s), 4.38 (1H, s), 4.33 (1H, s), 3.93–3.84 (2H, m), 3.86 (3H, s), 3.71–3.45 (41H, m), 2.62 (2H, s), 2.29 (1H, td, *J* = 13.7, 5.2 Hz), 2.21 (2H, br s), 2.04 (1H, d, *J* = 10.9 Hz), 1.98 (1H, d, *J* = 13.2 Hz), 1.80 (1H, d, *J* = 10.3 Hz), 1.66 (2H, br s), 1.58–1.21 (16H, m), 1.13 (3H, d, *J* = 5.7 Hz), 1.03 (3H, s), 0.93 (3H, d, *J* = 6.3 Hz), 0.88 (1H, d, *J* = 12.0 Hz), 0.85 (3H, s). MS (ESI-TOF) *m*/*z*: 1155 [M + Na]^+^. HRMS (ESI-TOF) *m*/*z*: 1155.6781 calcd for C_58_H_96_N_6_O_16_Na; Found: 1155.6776.

#### 3.3.5. Methyl 4-Hydroxy-3-(prop-2-yn-1-yloxy)-5-(((1*S*,2*R*,4a*R*,8a*R*)-1,2,4a-trimethyl-5-oxodecahydronaphthalen-1-yl)methyl)benzoate (**10**)

To a solution of **9** (41.6 mg, 0.11 mmol) in DMF (2.2 mL) was added K_2_CO_3_ (18.4 mg, 0.13 mmol) and propargyl bromide solution (80 wt % in toluene, 13 μL, 0.12 mmol) at 0 °C, and the whole mixture was stirred overnight at rt. The reaction was quenched by the addition of sat. NH_4_Cl aq. and the mixture was extracted with AcOEt. The combined organic phase was dried over Na_2_SO_4_, and the solvent was evaporated in vacuo. Purification by SiO_2_ column chromatography (*n*-hexane/AcOEt = 5:1 to 1:1) gave **10** (7.5 mg, 17%) as colorless amorphous solid.

^1^H-NMR (500 MHz, CDCl_3_) δ: 7.50 (1H, d, *J* = 1.5 Hz), 7.46 (1H, d, *J* = 1.5 Hz), 6.14 (1H, s), 4.80 (1H, s), 4.79 (1H, s), 3.87 (3H, s), 2.74 (1H, d, *J* = 14.1 Hz), 2.70 (1H, d, *J* = 14.1 Hz), 2.62–2.55 (2H, m), 2.25 (1H, d, *J* = 12.8 Hz), 2.17 (1H, m), 2.12–2.09 (1H, m), 1.80 (1H, qd, *J* = 13.0, 3.2 Hz), 1.69–1.61 (1H, m), 1.45–1.18 (5H, m), 1.15 (3H, s), 1.15–1.12 (1H, m), 1.03 (3H, d, *J* = 6.1 Hz), 0.94 (3H, s). ^13^C-NMR (125 MHz, CDCl_3_) δ: 216.4, 166.8, 149.5, 144.0, 128.2, 124.3, 120.6, 111.3, 77.4, 76.6, 57.3, 52.0, 49.2, 47.4, 42.4, 37.5, 36.9, 35.6, 32.3, 26.7, 25.3, 22.0, 18.9, 18.0, 17.4. IR (KBr): 3291, 2928, 1705, 1437, 1302, 1211 cm^−1^. MS (ESI-TOF) *m*/*z*: 435 [M + Na]^+^. HRMS (ESI-TOF) *m*/*z*: 435.2147 calcd for C_25_H_32_O_5_Na; Found: 435.2168.

#### 3.3.6. Methyl 4-Hydroxy-3-((1-(39-((4*R*,5*S*)-5-methyl-2-oxoimidazolidin-4-yl)-34-oxo-3,6,9,12,15,18,21,24,27,30-decaoxa-33-azanonatriacontyl)-1*H*-1,2,3-triazol-4-yl)methoxy)-5-(((1*S*,2*R*,4a*R*,8a*R*)-1,2,4a-trimethyl-5-oxodecahydronaphthalen-1-yl)methyl)benzoate (**5**, probe C)

To a solution of **10** (2.3 mg, 5.58 μmol) and **6** (5.2 mg, 7.25 μmol) in DMF (0.2 mL) was added CuI (0.3 mg, 1.67 μmol) and DIPEA (1.9 μL, 11.2 μmol), and the whole mixture was stirred for 16 h at rt. Concentration in vacuo gave a crude product, which was purified by SiO_2_ column chromatography (CHCl_3_/MeOH/H_2_O = 30:3:1, lower phase) to give **5** (3.7 mg, 58%) as colorless amorphous solid.

^1^H-NMR (500 MHz, CDCl_3_) δ: 7.92 (1H, s), 7.56 (1H, d, *J* = 1.7 Hz), 7.44 (1H, d, *J* = 1.7 Hz), 6.90 (1H, s), 6.24 (1H, br s), 5.26 (2H, s), 4.61 (1H, br s), 4.58 (2H, t, *J* = 4.9 Hz), 4.28 (1H,br s), 3.89 (2H, t, *J* = 5.2 Hz), 3.87 (3H, s), 3.83 (1H, t, *J* = 6.9 Hz), 3.71–3.59 (34H, m), 3.55 (2H, t, *J* = 4.9 Hz), 3.44 (2H, dd, *J* = 10.0, 5.4 Hz), 2.73 (1H, d, *J* = 14.3 Hz), 2.69 (1H, d, *J* = 14.3 Hz), 2.58 (1H, td, *J* = 14.0, 7.4 Hz), 2.24 (1H, d, *J* = 13.2 Hz), 2.20–2.16 (1H, m), 2.18 (2H, t, *J* = 7.4 Hz), 2.12–2.07 (1H, m), 1.79 (1H, qd, *J* = 12.9, 3.4 Hz), 1.52-1.18 (18H, m), 1.15 (3H, s), 1.12 (3H, d, *J* = 6.3 Hz), 1.02 (3H, d, *J* = 6.3 Hz), 0.93 (3H, s). MS (ESI-TOF) *m*/*z*: 1157 [M + Na]^+^. HRMS (ESI-TOF) *m*/*z*: 1157.6573 calcd for C_57_H_94_N_6_O_17_Na; Found: 1157.6576.

### 3.4. Binding Ability of Probes A–C (**3–5**) to Proteins in Cell Lysates

Cell lysates prepared from DU145 cells cultured under normoxic or hypoxic conditions (100 μg protein each) in 250 μL binding buffer (25 mM Tris-HCl (pH 7.6) containing 150 mM NaCl, 1 mM ethylenediaminetetraacetic acid (EDTA), 0.025% sodium dodecyl sulfate (SDS)), were incubated for 1 h in the presence of each probe (400 pM) with gentle rotation. After 1 h, the probes were pulled down by adding 200 μL of streptavidin-conjugated Dynabeads, and the resulting supernatants were moved to new test tubes. The Dynabeads were washed with binding buffer three times, and the resulting washed solutions were combined with the corresponding supernatants. These solutions were concentrated using Amicon Ultra 10-kDa centrifugal filter units (Millipore, Darmstadt, Germany) and used for western blot analysis to detect candidate proteins (EIF5AL1, MIA3, RBM28, RPAP3, and TRMT6). The Dynabeads were resuspended in 100 μL of 1× sample buffer for SDS-polyacrylamide gel electrophoresis (PAGE), boiled for 5 min at 95 °C, and centrifuged, and the supernatants were used for western blotting.

### 3.5. Western Blotting Analysis

DU145 cells or their transformants (5 × 10^5^ cells/2 mL/6-well plastic plate) were rinsed with ice-cold phosphate-buffered saline (PBS) and lysed in lysis buffer (25 mM Tris-HCl (pH 7.6) containing 150 mM NaCl, 1 mM EDTA, 1% NP-40, 0.1% SDS, and 1% protease inhibitor cocktail). Cell lysates were subjected to SDS-PAGE and transferred to polyvinylidene difluoride membranes (GE Healthcare Life Sciences, Buckinghamshire, UK). The membranes were then incubated with appropriate primary antibodies and HRP-conjugated secondary antibodies, and immunopositive bands were visualized using an enhanced chemiluminescence kit (GE Healthcare Life Sciences). The luminescent signals were analyzed using an ImageQuant LAS4010 Scanner (GE Healthcare Life Sciences).

### 3.6. Generation of RPAP3-Overexpressing DU145 Cells

In order to construct the RPAP3 expression plasmid (pEBMulti_RPAP3), cDNA encoding human RPAP3 was synthesized by FASMAC Co., Ltd. (Kanagawa, Japan) and ligated into pEBMulti-Neo (Wako Pure Chemical Industries), which is a mammalian expression vector, using *ClaI* and *EcoRI* restriction enzyme sites. Next, DU145 cells were transformed with 2.5 μg pEBMulti_RPAP3 using Lipofectamine LTX (Invitrogen) according to the manufacturer’s protocol. After transformation, DU145 cells were cultured in RPMI 1640 containing 10% FBS and 500 μg/mL G418 for 1 week. The surviving cells were used for the experiment as stable human RPAP3-overexpressing cells. In addition, the expression levels of RPAP3 were confirmed by western blotting.

### 3.7. Preparation of MIA3-, RBM28-, and RPAP3-Knockdown DU145 Cells

siRNAs for knockdown of MIA3, RBM28, and RPAP3 genes were synthesized by Cosmo Bio Co., Ltd. (Tokyo, Japan). The sequences of siRNAs used to knockdown *MIA3* were as follows: siRNA_MIA3_1, 5′-UUAACAUGCUGCAUUCGUCdTdT-3′; siRNA_MIA3_2, 5′-UUUUAAAAUUCACAAAACGdTdT-3′; and siRNA_MIA3_3, 5′-UUAUAGUAAACAUAUACAGdTdT-3′. The sequences of siRNAs used to knockdown *RBM28* were as follows: siRNA_RBM28_1, 5′-CAAGGAGAUUACCACCUUUdTdT-3′; siRNA_RBM28_2, 5′-GCUCAGAGUGAUACCAGCAdTdT-3′; and siRNA_RBM28_3, 5′-GAAGUAAAUAUCCCUAGGAdTdT-3′. The sequences of siRNAs used to knockdown *RPAP3* were as follows: siRNA_RPAP3_1, 5′-UCCAGAUAUGUUGUAUCAGdTdT-3′; siRNA_RPAP3_2, 5′-CUCUCCAAAAUUAAAAAGGdTdT-3′; and siRNA_RPAP3_3, 5′-UCCAGAUAUGUUGUAUCAGdTdT-3′. Each of the three siRNAs (20 nM each) for each gene were mixed and used for the experiment. DU145 cells (5 × 10^4^ cells/well) were precultured in RPMI 1640 supplemented with 10% FBS for 24 h, and cells were then transfected with 60 nM of each siRNA cocktail via the lipofection method using Gene Silencer (Genlantis Inc., San Diego, CA, USA) after exchange of the medium for RPMI 1640 without FBS. After incubation for 4 h, the cells were cultured in RPMI 1640 containing 20% FBS for 48 h and then used as gene-knockdown cells in the experiments. The expression levels of various proteins and the proliferation of each knockdown cell line were evaluated by western blotting and MTT assays.

### 3.8. Preparation of a Peptide-Display Phage Library and Screening of Probe A (**3**)-Binding Phages

Total RNA was isolated from DU145 cells cultured under hypoxic conditions using a RNeasy Plus Mini Kit (Qiagen), and mRNA was purified using an Oligotex-dT30 mRNA Purification Kit (Takara, Shiga, Japan) according to the manufacturer’s protocol. Next, purified mRNA (4 µg) was randomly primed using an Orient Express Random Primer cDNA Synthesis Kit (Merck) to yield double-stranded cDNA. The cDNA was added the directional *EcoRI*/*HindIII* linkers, size fractionated, and double-digested with *EcoRI* and *HindIII* endonucleases. The purified cDNA was then directionally ligated into T7Select1-2b vector arms and subsequently in vitro packaged into phagemids. The packaging mixture was evaluated to determine the ligation efficiency by infection of *Escherichia coli* BLT5615, and the ligation efficiency was calculated as 3 × 10^6^ pfu/mL. This initial phage solution was amplified by infecting *E. coli* BLT5615, and the phage library was obtained with an overall titer of 2 × 10^10^ pfu/mL.

In order to screen the probe A (**3**)-binding phage, 200 pmol probe A (**3**) was added to 1 mL (2 × 10^10^ pfu) of phage library solution (20 mM Tris-HCl (pH 8.0), 100 mM NaCl, 6 mM MgSO_4_) and incubated for 3 h at 4 °C with rotation. Subsequently, 100 µL streptavidin-conjugated Dynabeads were mixed with the phage library solution including probe A (**3**) and incubated for 1 h. The resulting beads were washed 10 times with PBS containing 0.1% Tween 20, and probe A (**3**)-binding phages were eluted using 100 µL PBS containing 1.0% Tween 20. Next, 5 µL elute was used for titering, and the remaining sample was used to infect *E. coli* BLT5615 for amplification. This selection cycle, called biopanning, was repeated seven times, and 30 individual phages were selected to analyze genomic DNA to identify the displayed peptide. Sequencing of isolated genomic DNA was performed by Macrogen Japan (Kyoto, Japan) with the primers T7-up (5′-GGAGCTGTCGTATTCCAGTC-3′) and T7-down (5′-AACCCCTCAAGACCCGTTTA-3′). The obtained sequences were analyzed by BLAST search with the UniProtKB/Swiss-Prot database.

The binding ability of probes A–C (**3**–**5**) to the phages was evaluated as the recovery rate of the phages. Each phage solution (1 × 10^9^ pfu/mL) was mixed with 200 pmol probes, and a single cycle of biopanning was performed. Then, the plaque-forming units (titers) of the eluate from Dynabeads was measured. The recovery rate was calculated as follows: titer of eluate/titer of initial phage solution × 100.

### 3.9. Statistical Analaysis

Data are shown as means ± standard errors of the means, and the differences between data sets were assessed by Dunnett’s test. Differences with *p* values of less than 0.05 were considered significant.

## 4. Conclusions

In this study, we aimed to identify target molecules (binding proteins) of dictyoceratin-A (**1**) and -C (**2**) as inhibitors of hypoxia-adapted cancer cells. To this end, we synthesized the three types of probe molecules (**3**–**5**), which showed different biological properties in DU145 cells under hypoxic and normoxic conditions. Then, the candidates for target molecules were identified from a peptide-displaying phage library utilizing these probe molecules. Finally, characterization studies of RPAP3-knockdown and -overexpressing DU145 cells revealed that RPAP3, a component of the R2TP/PEDL complex, was the target molecule of dictyoceratin-A (**1**) and -C (**2**). In addition, we proposed that decreased HIF-1α levels in DU145 cells treated with dictyoceratin-A (**1**) and -C (**2**) were related to inhibition of mTOR stabilization by the R2TP/PEDL/HSP90 complex (Figure 8). To date, no inhibitors of RPAP3 have been discovered. Thus, dictyoceratin-A (**1**) and -C (**2**) may have applications as new medicines for cancer chemotherapy and useful tools for analyzing the intracellular functions of the R2TP/PEDL/HSP90 complex.

## Supplementary Materials

The following are available online at https://www.mdpi.com/1660-3397/17/3/163/s1, Scheme S1: Synthesis of probe molecules **3**, **4**, and **5**.
