# Supplementary figures and images for: Target Identification of the Marine Natural Products Dictyoceratin-A and -C as Selective Growth Inhibitors in Cancer Cells Adapted to Hypoxic Environments
Source: Mar Drugs. 2019 Mar 8;17(3):163. doi: 10.3390/md17030163 (PMC6471994; doi:10.3390/md17030163)

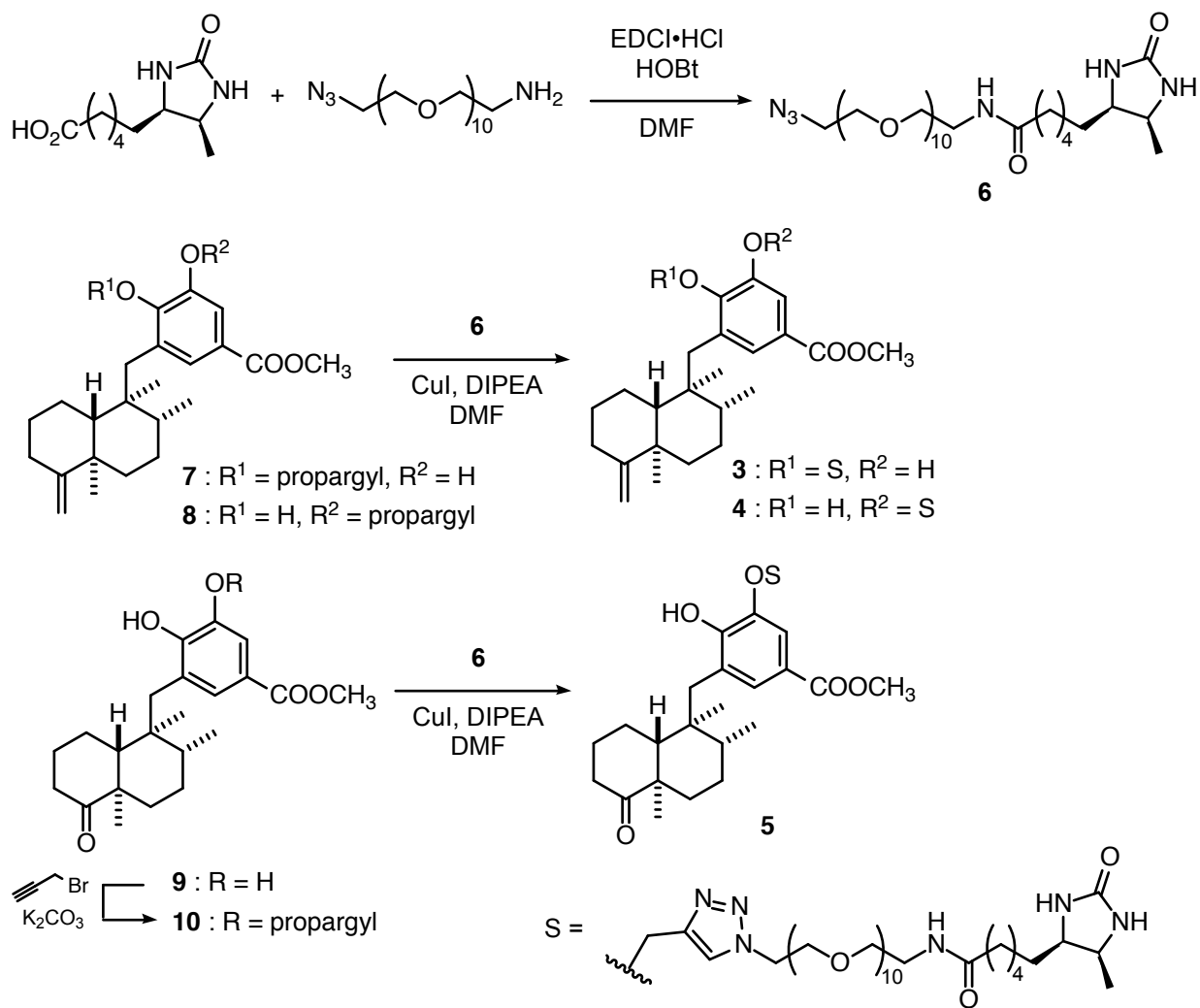

**Supplementary scheme S1.** Synthesis of probe molecules **3**, **4**, and **5**.

Supplement: Supplementary file 1 [file marinedrugs-17-00163-s001.pdf]
